# Impaired Healing of a Cutaneous Wound in an Inducible Nitric Oxide Synthase-Knockout Mouse

**DOI:** 10.1155/2017/2184040

**Published:** 2017-04-13

**Authors:** Takashi Kitano, Hiroshi Yamada, Maki Kida, Yuka Okada, Shizuya Saika, Munehito Yoshida

**Affiliations:** ^1^Department of Orthopedic Surgery, Wakayama Medical University School of Medicine, 811-1 Kimiidera, Wakayama 641-0012, Japan; ^2^Department of Ophthalmology, Wakayama Medical University School of Medicine, 811-1 Kimiidera, Wakayama 641-0012, Japan; ^3^Department of Critical Care Medicine, Wakayama Medical University School of Medicine, 811-1 Kimiidera, Wakayama 641-0012, Japan

## Abstract

*Background*. We investigated the effects of loss of inducible nitric oxide synthase (iNOS) on the healing process of cutaneous excisional injury by using iNOS-null (KO) mice. Population of granulation tissue-related cell types, that is, myofibroblasts and macrophages, growth factor expression, and reepithelialization were evaluated.* Methods*. KO and wild type (WT) mice of C57BL/6 background were used. Under general anesthesia two round full-thickness excision wounds of 5.0 mm in diameter were produced in dorsal skin. After specific intervals of healing, macroscopic observation, histology, immunohistochemistry, and real-time reverse transcription-polymerase chain reaction (RT-PCR) were employed to evaluate the healing process.* Results*. The loss of iNOS retards granulation tissue formation and reepithelialization in excision wound model in mice. Detailed analyses showed that myofibroblast appearance, macrophage infiltration, and mRNA expression of transforming growth factor b and of collagen 1*α*2 were all suppressed by lacking iNOS.* Conclusions*. iNOS is required in the process of cutaneous wound healing. Lacking iNOS retards macrophage invasion and its expression of fibrogenic components that might further impair fibrogenic behaviors of fibroblasts.

## 1. Introduction

Rapid and well-organized wound healing in skin is essential to health maintenance by reducing risks of bacterial contamination, inhibition of water loss, suppression of scar formation that might perturb organ function, and so forth. Therefore, the mechanism of skin wound healing is to be well-understood in order to develop strategies to overcome impaired wound healing in conditions such as pressure ulcer and diabetic wounds.

The process of cutaneous wound healing overall consists of stages of so-called hemostasis, proliferation, and maturation [1–3]. The stage of “hemostasis” is the first stage preventing excessive blood loss and the trigger events that lead to local inflammation by neutrophils and then macrophages. The inflammation is followed by the performance of local tissue cells, that is, keratinocyte and fibroblast. The former cells first migrate into the injured area for the primary coverage and start to proliferate to recover the stratification. The latter transforms to the myofibroblast that is capable of producing extracellular matrix (ECM) and of tissue contraction. Both cell migration of keratinocytes and fibroblast-myofibroblast conversion largely depend on the activity of a potent growth factor, transforming growth factor *β* (TGF*β*), although a set of growth factors are believed to orchestrate the whole process of tissue repair [3].

Nitric oxide (NO) is implicated in cellular and molecular events of aspects of wound healing, that is, vasodilation, angiogenesis, inflammation, tissue fibrosis, or immune responses [4–6]. These reports overall suggest NO synthesis is essential to the uncomplicated cutaneous wound healing. However, controversial results were reported in types of wound models [7, 8]. NO production is mediated by inducible nitric oxide synthase (iNOS) that is regulated independently of intracellular calcium elevations [9, 10]. Initial injury is followed by infiltration of inflammatory cells, that is, neutrophils and macrophages, fibroblast repopulation and its transformation to myofibroblast, and new vessel formation as well as keratinocyte migration and proliferation. The major source of TGF*β* in a tissue under repairing process is considered to be macrophage. Recruitment of macrophage to an injured tissue is stimulated by NO [11–13]. Moreover, NO reportedly modulates expression of TGF*β* [7]. It is therefore hypothesized that NO might affect the healing process of cutaneous injury.

To address this question, in the present study we took an advantage of the availability of a mouse line of C57BL/6 background that lacks iNOS gene in our laboratory. To clear a disadvantage of the qualitative assessment of the wound healing process in the skin, we investigated statistical analysis of the incidence of closure of circular excision injury in the dorsal skin of mice during healing intervals, employing macroscopic observation, histology, immunohistochemistry, and detection of mRNAs using real-time reverse transcription-polymerase chain reaction (RT-PCR). Our statistical analysis showed that primary healing of excision cutaneous injury is impaired by the loss of iNOS in association with suppression of macrophage population and fibrogenic gene expression.

## 2. Materials and Methods

Experiments were approved by the DNA Recombination Experiment Committee and the Animal Care and Use Committee of Wakayama Medical University.

### 2.1. Excision Wound Model in Mice

iNOS-knockout (KO) and wild type (WT) mice, both of C57BL/6 background and 8 to 10 weeks old, were used in the experiments. Under general anesthesia with intraperitoneal administration by pentobarbital sodium (70 mg/kg of body weight) [14, 15], the dorsal hairs were shaved and the exposed skin was wiped with 70% ethanol. Two round full-thickness excision wounds of 5.0 mm in diameter were produced in dorsal skin by picking up a fold skin at the midline and using a sterile disposable biopsy trephine (Kai Industries, Gifu, Japan) and a surgical blade as previously reported by us [16]. The wounds arranged lengthways were made at the same time. Ofloxacin ointment (0.3%) was topically applied to the wounds to reduce the risk of bacterial contamination every day during the first 7 postwounding days. The mouse with an apparent sign of infection was excluded.

### 2.2. Macroscopic Wound Observation

Six KO and 6 WT mice were used for the analysis in macroscopic findings. The wound was photographed with SZ-PT (Olympus, Tokyo, Japan) at intervals of healing (0, 1, 3, 6, 8, 11, and 14 days). The size of remaining skin defect (as the percentage of the initial wound area) was evaluated by using Photoshop software (Version 8.0, Adobe Systems, Tokyo, Japan) and statistically analyzed.

### 2.3. Histology of Healing Tissue

Thirty-two KO and 32 WT mice were killed at specific intervals of healing (3, 6, 8, 11, and 14 days) with an overdose of diethyl ether and processed for histology. Two round excision wounds including the epithelial margins were excised 5.0 mm in diameter with a sterile disposable biopsy trephine and a surgical blade. The tissue excised wound was fixed with 4.0% paraformaldehyde in 0.1 M phosphate buffer (pH 7.4) for 48 hours and embedded in paraffin and processed for histology. Paraffin sections 5 *μ*m thick were cut and stained with hematoxylin and eosin (HE). We measured the thickness of granulation tissue at the two following points of the healing wound; one point was the center zone with the minimum thickness of the granulation tissue; another was the margin zone with the maximum thickness of the granulation tissue [17]. These data of the thickness of granulation tissue were statistically analyzed. Masson-Goldner trichrome stain was also performed for the visualization of collagenous connective tissue in the regenerated healing tissue.

### 2.4. Reepithelialization in Excisional Skin Wound Healing

We further analyzed the reepithelialization in excision skin wound healing with KO and WT mice at specific intervals of healing at the histological level (3, 6, 8, 11, and 14 days). For this purpose we used tissue stained with Masson-Goldner trichrome. The percentage of the reepithelialization to the original wound area was calculated as follows: reepithelization (%) = [distance covered by epithelium] × 100/[distance between original wound edge] [18–21].

### 2.5. Immunohistochemistry

Immunohistochemical analysis was performed for the evaluation of the cellular components in the regenerated tissue. Deparaffinized sections were processed for indirect immunohistochemistry as previously reported [22, 23]. The following antibodies were used [22, 23]; the presence of monocytes/macrophages was examined by using rat monoclonal F4/80 anti-macrophage antigen antibody (Clone A3-1, 1 : 400; BMA Biomedicals, August, Switzerland). The mouse monoclonal anti-*α*-smooth muscle actin (*α*SMA) antibody (Clone 1A4, 1 : 100; Neomarkers, Fremont, CA) was used to detect myofibroblast. Two visual fields (×400) were chosen from each edge of the wound bed; the other three were chosen from the middle of the wound bed at 6 and 8 days after the injury. The numbers of F4/80-positive macrophages and myofibroblasts as detected by *α*SMA expression within a wound bed were enumerated on these five visual fields [24, 25].

### 2.6. Detection of mRNAs Using Real-Time Reverse Transcription-Polymerase Chain Reaction (RT-PCR)

Two round wounds of 5.0 mm in diameter were produced using trephine and a blade in dorsal skin of each of 24 KO and 24 WT mice. For extraction of RNA, a round newly healed tissue was excised using trephine and a blade with the same size as the original wound at days 3, 6, and 8 and stored at deep freezer (−80°C). Total RNA was obtained using Sigma GenEluted™ Mammalian Total RNA Miniprep Kit (Sigma-Aldrich Co., St. Louis, MO) as previously reported [26–28]. Expression of mRNAs of *α*SMA, F4/80, transforming growth factor *β*1 (TGF*β*1), and collagen 1*α*2 in newly regenerated tissue was evaluated by RT-PCR. The TaqMan one-step RT-PCR master mix reagents kit and the Applied Biosystems Prism 7700 (PE Applied Biosysems, Foster City, CA) were used as described previously [26–28]. Primers and oligonucleotide probes were designed according to the cDNA sequences in the GenBank database using the Primers Express software (PE Applied Biosystems).

### 2.7. Statistical Analysis

The means and standard deviations were calculated for all parameters determined in this study. Statistically significant difference was evaluated by using unpaired Student's *t*-test. *p* < 0.05 was accepted as statistically significant.

## 3. Results

### 3.1. Macroscopic Findings of Wound Healing

**Figure 1:**
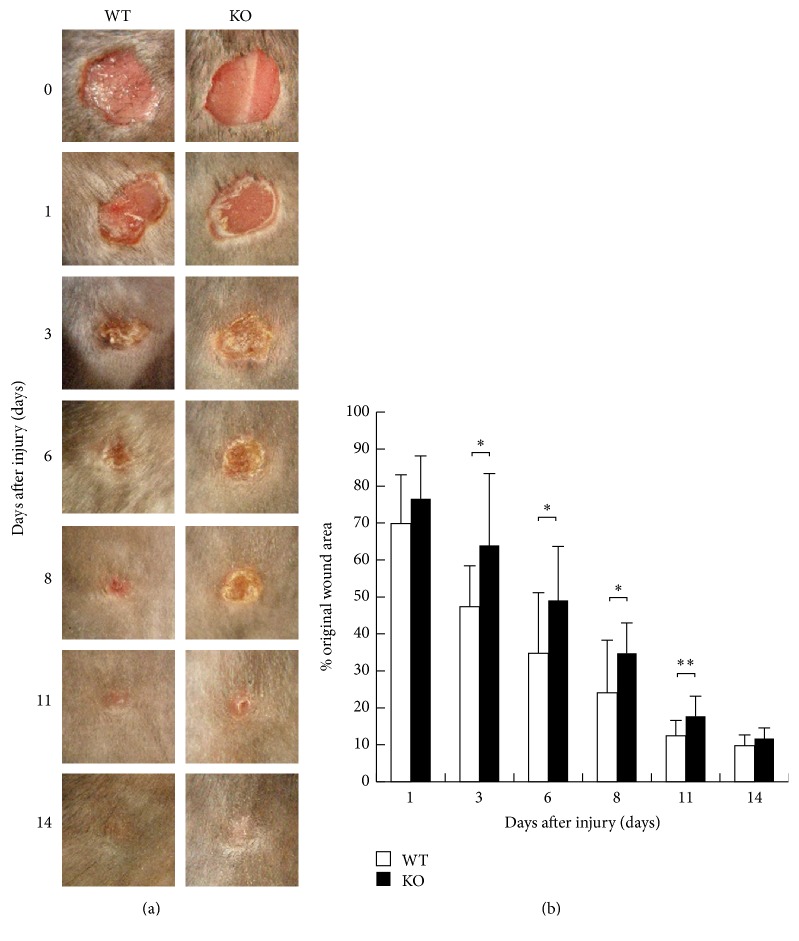
(a) Macroscopic wound findings of cutaneous excision wound healing in dorsal skin of inducible nitric oxide synthase (iNOS-) knockout (KO) mice and wild type (WT) mice. (b) Unhealed wound area changed in percentage to the original wound area in each time point. At the time points of 3, 6, 8, and 11, the remaining skin defect was larger in KO mice than WT mice with a statistically significant difference. Open bars: WT, filled bars: KO (*n* = 7 animals in each group). Mean ± standard deviation. ^*∗*^*p* < 0.05; ^*∗∗*^*p* < 0.01.

To evaluate the healing process of the dorsal excision wounds of KO and WT mice, we examined the size of remaining skin defect areas (as percentage of the original wound size) at each healing interval (Figure 1(a)). One day after injury, skin defects of KO mice were of similar morphology compared with WT mice. At the time points of 3, 6, 8, and 11, the remaining skin defect was larger in KO mice than WT mice with a statistically significant difference (Figure 1(b)). In WT mice, wound areas were reduced to 50% of the original wound areas at day 3 after injury. In contrast, wound areas in KO mice still remained at 50% even at day 6 after injury. At day 14 after the injury, cutaneous defects had no such difference. These observations demonstrated that healing of a full-thickness cutaneous wound was delayed in the absence of iNOS.

### 3.2. Histological Analysis of Granulation Tissue Formation

**Figure 2:**
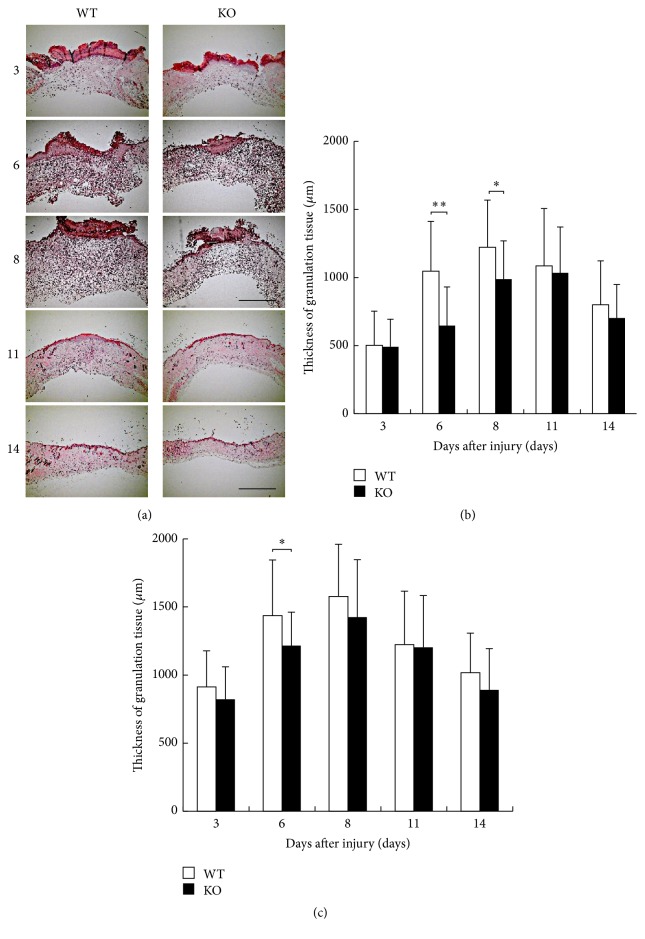
(a) Histology of healing wound tissue by hematoxylin and eosin stain at the indicated time intervals in dorsal skin of inducible nitric oxide synthase (iNOS-) knockout (KO) mice and WT mice. (b) The thickness of the granulation tissue at the center zone was thinner in KO mice than in WT mice at days 6 and 8 with a statistically significant difference. (c) At the margin zone, the thickness of the granulation tissue was significantly thinner in KO mice than in WT mice at day 6. Open bars: WT, filled bars: KO (days 3, 11, and 14: *n* = 6; days 6, 8: *n* = 7 animals in each group). Mean ± standard deviation. ^*∗*^*p* < 0.05; ^*∗∗*^*p* < 0.01; bar, 1 mm.

The defect of dermis was gradually closed by newly formed granulation tissue that was covered with epidermis during skin healing process. For this purpose we employed histological observation by HE staining (Figure 2(a)). We measured the thickness of granulation tissue at the center and margin zone in the healing wound as previously reported by us. The results showed that the thickness of the granulation tissue at the center zone was thinner in KO mice than in WT mice at days 6 and 8 with a statistically significant difference (Figure 2(b)). At the margin zone, the thickness of the granulation tissue was significantly thinner in KO mice than in WT mice at day 6 (Figure 2(c)).

### 3.3. Evaluation of Reepithelialization

**Figure 3:**
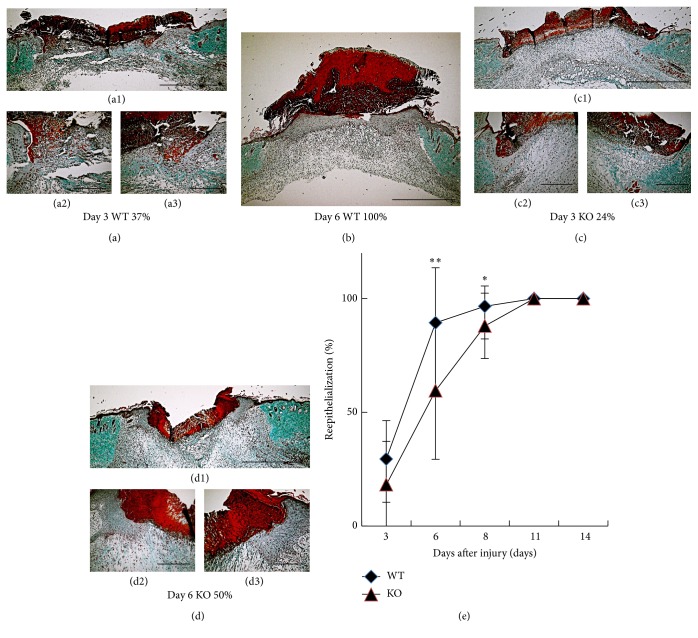
(a–d) Histological observation of reepithelialization of skin wound in wild type (WT) ((a1)–(a3), b) and inducible nitric oxide synthase (iNOS-) knockout (KO) ((c1)–(c3), (d1)–(d3)) mice at day 3 ((a1)–(a3), (c1)–(c3)) and day 6 (b, (d1)–(d3)) after injury. Masson-Goldner trichrome stain was viewed at low and high magnification to measure the original wound distance and reepithelialization area, respectively. (e) The ratio of reepithelialization was evaluated. Reepithelialization was significantly delayed in KO mice at days 6 and 8 with statistical difference compared with WT mice. ◆: WT, ▲: KO (days 3, 11, and 14: *n* = 6; days 6, 8: *n* = 7 animals in each group). Mean ± standard deviation. ^*∗*^*p* < 0.05; ^*∗∗*^*p* < 0.01; bar, 1 mm (a1, b, c1, and d1), 200 *μ*m ((a2)-(a3), (c2)-(c3), and (d2)-(d3)).

Newly formed granulation tissue in full-thickness cutaneous wound is associated with reepithelialization by keratinocytes. We evaluated the reepithelialization in skin wound healing in KO and WT tissues stained with Masson-Goldner trichrome at specific intervals of healing (Figures 3(a)–3(d)). At day 1 after injury, regeneration of stratified epithelium could not be observed in both WT and KO mice (data was not shown). At 3 days after injury, there seemed to be no difference of the process of reepithelialization between KO and WT mice. Reepithelialization was significantly delayed in KO mice at days 6 and 8 with statistical difference compared with WT mice (Figure 3(e)). The original wound was covered completely with epithelium in day 11 in both WT and KO mice. The results suggested that loss of iNOS retards reepithelialization by keratinocytes in the process of healing of an excision cutaneous injury.

### 3.4. Immunohistochemical Analysis

**Figure 4:**
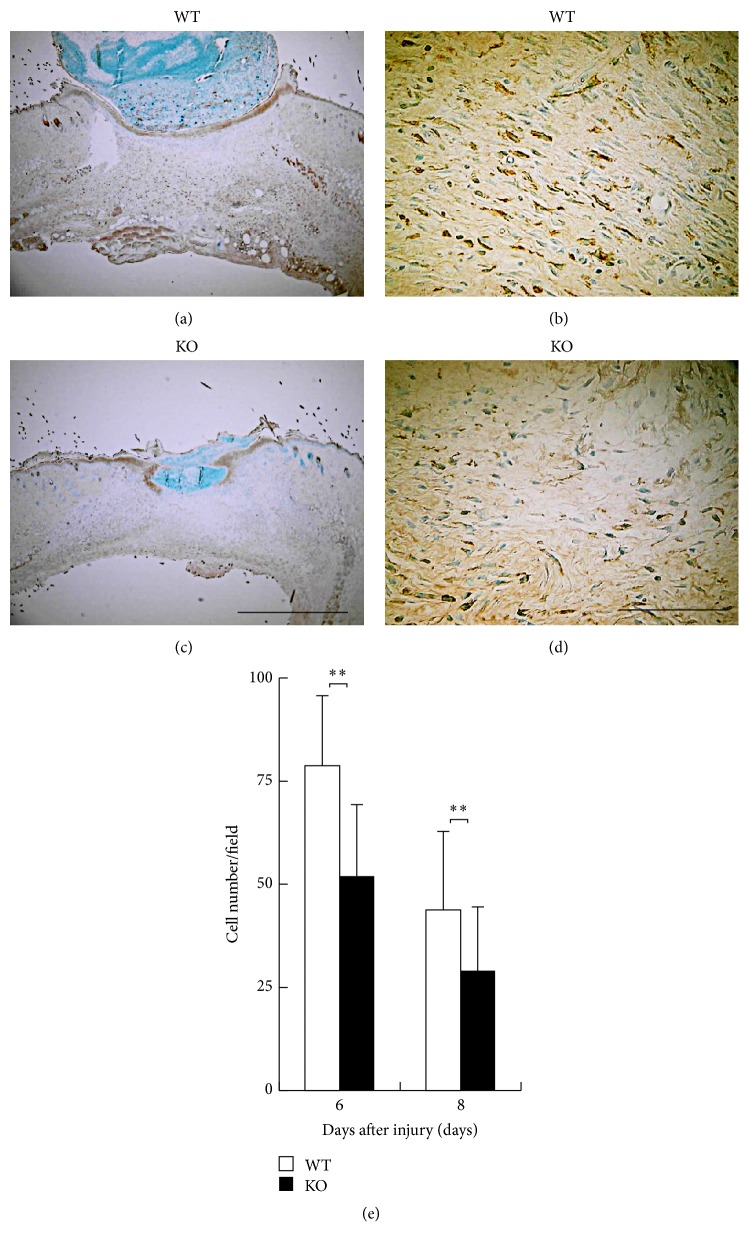
(a–d) Immunohistochemical observation of F4/80-labeled macrophages. Skin wound samples of wild type (WT) (a, b) and inducible nitric oxide synthase (iNOS-) knockout (KO) (c, d) mice indicate findings at day 6 after injury. (e) The numbers of F4/80-positive macrophages within a wound bed were enumerated on these five visual fields and analyzed macrophage recruitment. The infiltration of F4/80 labeled macrophages in KO mice was reduced with a statistical significance compared with WT mice at days 6 and 8 after injury. Open bars: WT, filled bars: iKO (days 6, 8: *n* = 7 animals in each group). Mean ± standard deviation. ^*∗∗*^*p* < 0.01; bar 10 *μ*m.

The nature of cellular components in the new granulation tissue was evaluated by immunohistochemistry. Although inflammatory cell types infiltrate into the healing tissue, macrophages reportedly play critical roles in the formation of granulation tissue or neovascularization via expression of growth factors.  Figure 4 indicates the immunohistochemical detection of F4/80-labeled macrophages at day 6. The infiltration of macrophages in KO healing tissue was reduced as compared with WT mice at days 6 and 8 after injury with a statistical significance being compared (Figure 4(e)).

**Figure 5:**
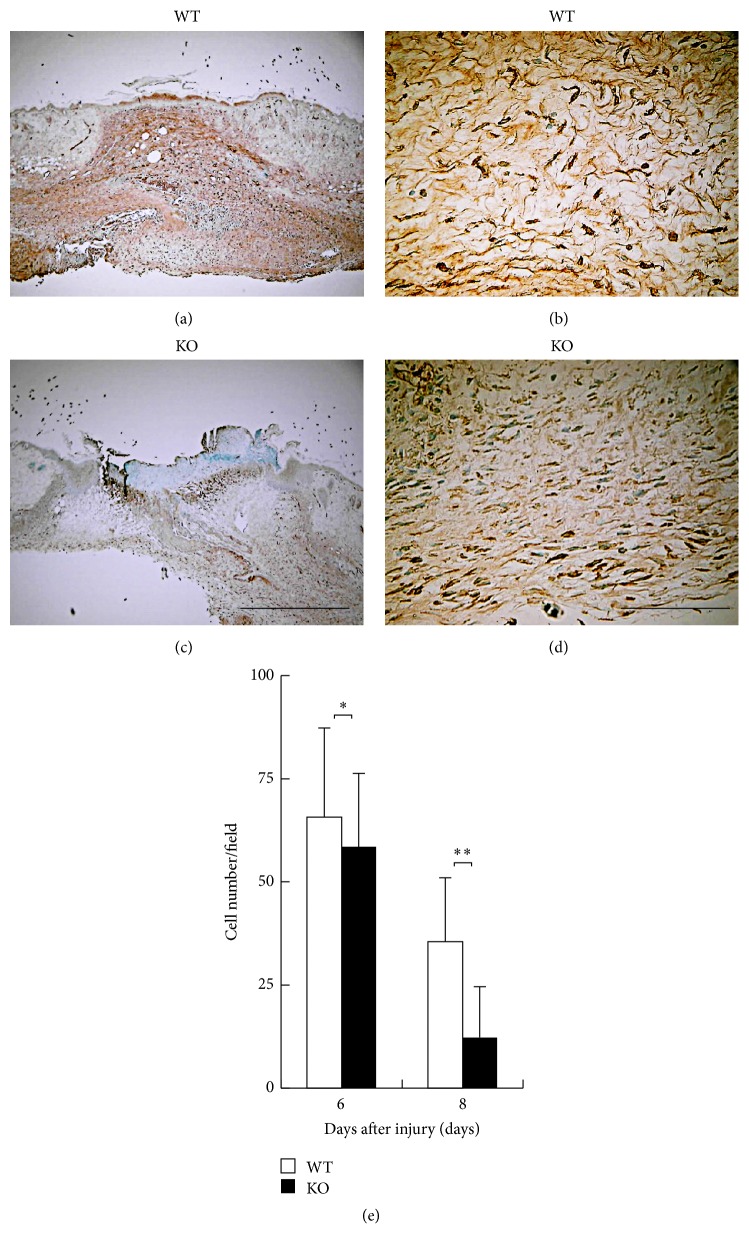
(a–d) Immunohistochemical observation of myofibroblasts as detected by *α*-smooth muscle actin (*α*SMA). Skin wound samples of wild type (WT) (a, b) and inducible nitric oxide synthase (iNOS-) knockout (KO) (c, d) mice indicate findings at day 6 after injury. (e) The numbers of myofibroblasts within a wound bed were enumerated on these five visual fields and analyzed. The appearance of myofibroblasts as detected by *α*SMA in KO mice was reduced with a statistical significance compared with WT mice at days 6 and 8 after injury. Open bars: WT, filled bars: KO (days 6, 8: *n* = 7 animals in each group). Mean ± standard deviation. ^*∗*^*p* < 0.05; ^*∗∗*^*p* < 0.01; bar 10 *μ*m.

Figure 5 shows the immunohistochemical distribution of myofibroblasts as detected by *α*SMA expression at day 6. Population of myofibroblasts was suppressed by the loss of iNOS at days 6 and 8 after injury as compared with WT mice with a statistical significance (Figure 5(e)). These results suggested that inhibition of macrophage invasion and myofibroblast appearance could explain the attenuation of granulation tissue formation and resultant epithelial resurface in KO healing tissue.

### 3.5. mRNA Expression of Normal Skin of WT and KO Mice

**Figure 6:**
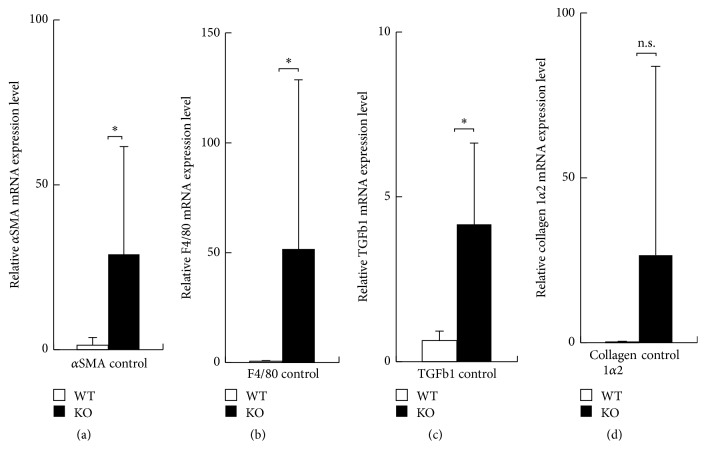
Expression of mRNA of *α*-smooth muscle actin (*α*SMA), F4/80, transforming growth factor *β*1 (TGF*β*1), and collagen 1*α*2 in normal skin of wild type (WT) and inducible nitric oxide synthase (iNOS-) knockout (KO) mice. (a–c) Expression of *α*SMA, F4/80 and TGF*β*1 mRNA was significantly more marked in KO normal skin compared with WT tissues. (d) Expression level of collagen 1*α*2 mRNA was more prominent in KO normal skin, but there was no significant difference between WT and KO. Open bars: WT, filled bars: KO. Mean ± standard deviation. ^*∗*^*p* < 0.05; n.s., not significant.

We examined the basal level of expression of *α*SMA, F4/80, TGF*β*1, and collagen 1*α*2 by performing real-time RT-PCR in cutaneous tissue of KO and WT mice. Although variation of the data among each animal seemed larger, the data indicated obvious difference between WT and KO mice. Expression of *α*SMA, F4/80, and TGF*β*1 mRNA was significantly more marked in KO normal skin tissue compared with WT tissues (Figures 6(a)–6(c)). There was no significant difference of the expression level of collagen 1*α*2 mRNA between KO and WT tissues (Figure 6(d)).

### 3.6. Gene Expression in Newly Regenerated Tissue of a Healing Wound

**Figure 7:**
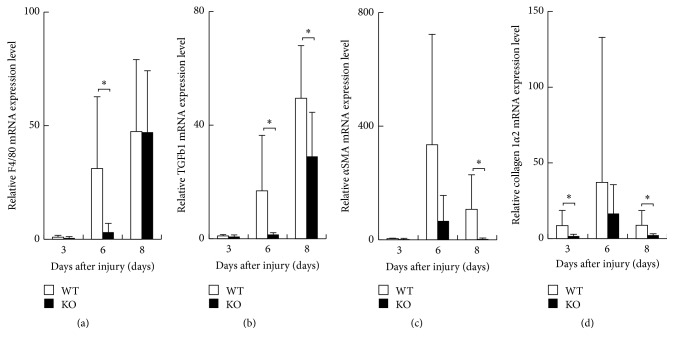
Expression of mRNA of *α*-smooth muscle actin (*α*SMA), F4/80, transforming growth factor *β*1 (TGF*β*1), and collagen 1*α*2 in healing tissues of wild type (WT) and inducible nitric oxide synthase (iNOS-) knockout (KO) mice at days 3, 6, and 8. (a) F4/80 mRNA expression was significantly more suppressed in KO tissues at day 6 as compared with WT tissues. (b) Expression of TGF*β*1 mRNA was significantly attenuated in KO tissues as compared with WT tissues at days 6 and 8. (c) Expression of *α*SMA mRNA was significantly less marked in KO tissues at day 8 as compared with WT tissues. (d) Collagen 1*α*2 mRNA expression was reduced in KO tissues as compared with WT tissues at days 3 and 8 with statistically significant difference. Open bars: WT, filled bars: KO. Mean ± standard deviation. ^*∗*^*p* < 0.05; n.s., not significant.

We then evaluated the expression pattern of mRNA of F4/80, TGF*β*1, *α*SMA, and collagen 1*α*2 in healing tissues of KO and WT mice at days 3, 6, and 8 by using real-time RT-PCR. F4/80 mRNA expression increased during healing process in both genotypes of mice. Although variation of the data among each animal seemed larger, the data indicated obvious difference between WT and KO mice. Expression of these components was significantly suppressed in KO tissues as compared with WT tissues at day 6 (Figure 7(a)). Expression of TGF*β*1 mRNA also increased in healing tissue and was significantly less in KO tissues as compared with WT tissues at days 6 and 8 (Figure 7(b)). Expression of *α*SMA mRNA was significantly less marked in KO tissues as compared with WT tissues at day 8 (Figure 7(c)). Collagen 1*α*2 mRNA expression was reduced in KO tissues as compared with WT tissues at days 3 and 8 with statistically significant difference (Figure 7(d)).

## 4. Discussion

In the present study, we showed that the healing of a circular excision wound in the dorsal skin was significantly impaired by the loss of iNOS in mice. Histology indicated that the formation of granulation tissue and reepithelialization were both attenuated in KO mice as compared with WT mice at each specific time point. Wound NO synthesis inhibition by blocking NOS activity or gene knockout reportedly impairs wound healing, in particular, collagen synthesis [7, 8, 29–31], that coincides with the current study.

Our current immunohistochemical analysis and real-time RT-PCR further confirmed the histology findings; invasion of macrophages and myofibroblast appearance were both suppressed by lacking iNOS. Macrophages reportedly play a critical role in the cutaneous healing by expressing a set of growth factors including TGF*β* [32]. Although various growth factors are involved in the process of tissue repair, TGF*β* is one of the most important ligands involved in the regulation of cell behavior in physiological or pathological processes of tissue repair [33]. Present study showed that expression of TGF*β*1 mRNA was significantly attenuated in KO healing tissues as compared with WT tissues at days 6 and 8. Previous studies suggest that NO is involved in migration and activation of TGF*β*1 of macrophages [34] that coincides with the present findings. Appearance of myofibroblasts and accumulation of provisional extracellular matrix is gradually replaced with a collagenous matrix, presumably as a result of the action of TGF*β*1 [35, 36]. Real-time RT-PCR also indicated that the mRNA expression of collagen 1*α*2 was less in KO mouse tissues as compared with WT tissues at days 3 and 8 after injury. A previous report indicated that iNOS-null fibroblasts proliferated more slowly, synthesized less collagen, and contracted fibroblast-populated collagen lattices more slowly than WT fibroblasts [4]. Reduction of tissue TGF*β*1 level could explain the reduction of myofibroblast appearance and collagen expression. However, in the uninjured tissue expression level of TGF*β*1, *α*SMA, F4/80, and collagen I was higher in a KO mouse as compared with a WT tissue. Detailed mechanism that could explain the phenomena was to be uncovered.

Epithelial cell migration is positively modulated by TGFb/p38 signal [37]. Reduced level of TGFb1 in tissue could explain the attenuation of epithelial healing in the absence of iNOS. Although cutaneous injury is well-repaired in normal subjects, impaired healing in diabetic patients or pressure ulcer is to be overcome. Reduction of NO level in tissue is associated with such attenuation of tissue repair conditions. Strategy to supply NO is considered to be one of the efficient ones in the treatment of impaired cutaneous wound healing.

## 5. Conclusion

The present study indicates that iNOS is required in the process of cutaneous wound healing. Lacking iNOS retards macrophage invasion and its expression of fibrogenic components that might further impair fibrogenic behaviors of fibroblasts.
